# Oral Health-Related Quality of Life in Dutch Children Diagnosed with Oligodontia. A Cross-Sectional Study

**DOI:** 10.3390/ijerph16132371

**Published:** 2019-07-04

**Authors:** Marieke A.P. Filius, Marco S. Cune, Marijn Créton, Arjan Vissink, Gerry M. Raghoebar, Anita Visser

**Affiliations:** 1 Department of Oral and Maxillofacial Surgery, University Medical Center Groningen and University of Groningen, PO Box 30.001, 9700 RB Groningen, The Netherlands; 2 Department of Restorative dentistry and Biomaterials, Center for Dentistry and Oral Hygiene, University Medical Center Groningen and University of Groningen, PO Box 30.001, 9700 RB Groningen, The Netherlands; 3 Department of Oral and Maxillofacial Surgery, Prosthodontics and Special Care, University Medical Center Utrecht and Utrecht University, PO Box 85090, 3854 EA Utrecht, The Netherlands

**Keywords:** hypodontia, oligodontia, oral health-related quality of life, children, orthodontics

## Abstract

There is need to get insight into condition-specific oral health-related quality of life in Dutch children with oligodontia. Between October 2014 and March 2017, 11–17-year-old oligodontia patients were approached to join a study assessing the impact of oligodontia on condition-specific oral health-related quality of life (OHrQoL). The patients received a condition-specific OHrQoL questionnaire prior to the start of orthodontic treatment. Non-oligodontia children in the same age group, but also requiring orthodontic treatment, were approached to serve as a control. The Fisher’s Exact Test was used for comparison purposes with the control group because of the small group sizes. Furthermore, subgroup analyses were performed for gender, age, number of congenitally missing teeth, tooth agenesis in the aesthetic region, orthodontic classification and microdontia via independent *t*-tests. *p*-values of <0.05 were considered statistically significant. Twenty-eight oligodontia patients and 23 controls agreed to participate. The oligodontia patients’ scores were comparable to the controls except for the items about dental appearance and treatment complexity. The impact of oligodontia on OHrQoL in youngsters is limited and mainly concerns dental appearance and the complexity of the treatment.

## 1. Introduction

Hypodontia is a condition in which one or more teeth are missing as a consequence of tooth agenesis. The term oligodontia is generally used when six or more teeth (third molars excluded) are congenitally absent [1]. As a consequence of missing teeth, children with oligodontia can develop functional or aesthetic problems that may result in a physical and emotional burden, especially during the turbulent years of adolescence [2].

Having congenitally missing teeth is likely to have an impact on perceived oral health-related quality of life (OHrQoL) in children [3,4]. However, it was reported that the OHrQoL in patients with congenitally missing teeth is comparable to that of routine orthodontic controls [5]. Thus, the impact of congenitally absent teeth on the children’s OHrQoL requires further verification, also because the OHrQoL has only been reported on the basis of non-condition specific questionnaires [3,4,5].

Several non-condition-specific questionnaires have been developed to asses OHrQoL in youngsters, such as: the Child Oral Health Quality of Life questionnaire (COHQoL [6]); the Child Oral Health Impact Profile (COHIP [7]); the Child-Oral Impacts on Daily Performances (Child-OIDP [8]); the Early Childhood Oral Health Impact Scale (ECOHIS [9]); and the Scale of Oral Health Outcomes (SOHO-5 [10]). The COHQoL questionnaire consists of two age-dependent Child Perceptions Questionnaires, viz. for 8–10 year olds (CPQ8–10) and for 11–14 year olds (CPQ11–14), which restricts its use in prospective studies [11]. The CPQ has been used to investigate the quality of life in children with congenitally missing teeth [3,4,5]. However, the CPQ and the other aforementioned questionnaires have one thing in common and that is they were developed to measure OHrQoL in children who are not diagnosed with oligodontia, cleft palate or other craniofacial afflictions.

As the management of oligodontia is a complex process and is likely to differ from children not diagnosed with oligodontia, there is a need for a condition-specific OHrQoL questionnaire [12]. Akram et al. tried to bridge that omission by developing a condition-specific quality-of-life-in-children-with-developmentally-absent-teeth (ChildQoLDAT) measure for children between 11 and 18 years of age [12,13]. Oligodontia condition-specific problems can be recognized and understood better with the items of this questionnaire, yet it has never been used to describe the differences in OHrQoL between children with oligodontia and unaffected controls. Moreover, the potential differences in OHrQoL between primary school children (≤12 years) and secondary school children (≥13 years) is interesting as appearance possibly becomes more important during puberty and adolescence.

The aim of this study was to assess the condition-specific OHrQoL in 11–17-year-old Dutch oligodontia children compared to non-affected controls that require also orthodontic treatment. Based on literature, it was presumed that the OHrQoL of patients with oligodontia could be affected negatively as a result of the missing teeth compared to non-affected controls without congenitally missing teeth. The results of this study will help to elucidate how health care providers can enhance the OHrQoL more effectively [14]. None of the patients and controls had been treated orthodontically at the time of completing the OHrQoL questionnaire.

## 2. Materials and Methods

### 2.1. Patient Selection

Between October 2014 and March 2017, patients diagnosed with oligodontia who were referred to Dutch centers for special dental care (University Medical Center Groningen (UMCG) and University Medical Center Utrecht (UMCU) were considered eligible for the study) when they fulfilled the following criteria:

- 11–17 years of age;
- Agenesis of ≥6 teeth, excluding the third molars (oligodontia);
- Absence of a syndrome or a mental handicap;
- Patients are at the start of their orthodontic treatment, prior to fitting orthodontic appliances.

Counting the congenitally absent teeth on the panoramic radiograph made the diagnosis of oligodontia. This was done by the prosthodontist and/or the orthodontist. With regard to any prosthetic treatment, most of the patients still functioned well with their primary teeth and only when needed patients received pre-orthodontic prosthetic treatment. Regarding the control group, children between 11 and 17 years of age without any congenitally missing teeth, who were referred to the orthodontic department of the University Medical Center Groningen, were selected and approached prior to the start of their orthodontic treatment. The parents/caregivers of the patients and controls were informed about the study. All the included patients and controls received a questionnaire by regular mail. The patients and controls had to complete the questionnaire at home and send it back with a postage paid response envelope. We requested the patients to complete the questionnaire without any help. When the survey was not returned within 4 weeks, the parents/caregivers of the patient were called by phone and requested to return it as soon as possible. The Groningen medical ethical committee was approached for permission, but an exemption was granted as this research was an evaluation of routine dental care (M13.146644).

All the procedures performed in the studies involving human participants were in accordance with the ethical standards of the institutional and/or national research committee and with the 1964 Helsinki declaration and its later amendments or comparable ethical standards.

Informed consent was obtained from all individual participants included in the study.

### 2.2. Questionnaire

For this purpose, the items of the ChildQoLDAT, developed by Akram et al., were translated into Dutch [12,13]. The Guillemin et al. [15] and Wink et al. [16] translation approach was used on the ChildQoLDAT for the Dutch setting. Before the translation process was started, the first impression of the questionnaire (i.e., face validity) was determined as appropriate by the present authors [17]. First, the questionnaire was translated by a bilingual expert panel (4 male, 4 female; age mean 35 years, range 12–55 years; 3 persons with dental education, 5 persons with non-dental education). The bilingual expert panel included an English teacher (Language Center, University of Groningen) to secure the exact meaning of the English words, and a young woman of 12 years of age to check the understandability of the translations for the age category of the target audience (11–17 years old). Subsequently, a layman panel consisting of 3 young females without oligodontia (mean age 11 years, range 9–12 years), supported by two parents, judged the understandability of the Dutch translation of the questionnaire. For example, the Dutch translation of ‘contact sport’ was replaced with ‘sport’. Both panels were chaired by the same chairperson (MF), who led the discussion. Finally, a back-translation was performed by a native English person to evaluate whether or not both versions were compatible. Some words of the back-translation were not similar to the original; however, it was concluded that they were appropriate synonyms (e.g., affects (original) versus influences (back-translation)). The translation of ‘completely (dis)agree’ (Dutch: Helemaal (niet) mee eens) was changed in ‘strongly (dis)agree’ (Dutch: heel erg (niet) mee eens). Five children with oligodontia (1 male; 4 females; mean age 12 years, range 11–13 years), who met the inclusion criteria, were asked (with permission of their parents) to complete the current measure. The interviewer (M.F.) observed the children during the completion of the questionnaire and also focused on measure problems, relevance and missing elements. Afterwards, these children were interviewed individually to determine the face and content validity. Content validity examines the extent to which the concepts of interest are comprehensively represented by the items in the questionnaire [17]. The mean time for completing the questionnaire was 8.4 min. All five children considered the questionnaire to be understandable for their peers of the same age and they agreed that there were no missing items (the face- and content validity was high). The following adjustments were made. The words ‘day’, ‘month’ and ‘year’ were added to fill in the day of birth. ‘Family’ (Dutch: familie) replaced ‘parents/caregivers’ (Dutch: ouders/verzorgers) (items 5h and 5i). For four children some confusion arose regarding items about the orthodontic treatment because the treatment had not yet commenced. As a consequence, for item 2b the answer option ‘treatment is not yet started’ and for item 2f ‘I do not know if I will have to wear a false tooth’ were added.

This questionnaire includes 40 items and a graded response scale (1–5) is used for most of them, ranging from strongly agree to strongly disagree [12]. A low score represents a poor oral health-related quality of life. The same translated questionnaire was given to the oligodontia and control patients.

### 2.3. Statistics

The returned questionnaires were screened for missing values and/or items, which were noted as ‘not applicable’. When ≥15% of the patients had not answered a specific item, it was presumed that this item was either poorly understood or not applicable and the item was excluded from further analysis. When <15% of the patients did not answer a specific item, the missing value was filled in with the median of the group.

The scores of the relevant items of both the oligodontia and control groups were compared using the Fisher’s Exact Test (*p* = 0.05) because of the small group sizes. Differences in total score were calculated for the following factors: gender (male/female), age (11–12 versus 13–17 years), number of congenitally missing teeth (6–9 versus ≥ 10), tooth agenesis in the aesthetic region (no missing anterior teeth versus missing anterior teeth), orthodontic classification (class I/II/III) and microdontia (no microdontia versus microdontia). The Shapiro-Wilk test was used to analyze the normality of the total score of all the included graded response scale items. Independent *t*-tests and one-way ANOVA (Analysis of Variance) were used on the normally distributed data. When the data were not normally distributed, the Mann-Whitney U test was applied to test for statistical significance differences. *p*-values <0.05 were considered statistically significant (IBM SPSS Statistics 23, IBM Corp. Released 2015. IBM SPSS Statistics for Windows, Version 23.0. IBM Corp: Armonk, NY, USA).

## 3. Results

### Patients

Of the 39 selected oligodontia patients who met the inclusion criteria, 28 (72%) patients (and their parents/caregivers) agreed to participate; 19 from the UMCG and 9 from the UMCU. The other eleven patients did not return the questionnaire after several requests. The median number of congenitally missing teeth was 10 (IQR (interquartile range) [7;12]; range 6–18). Twenty-two patients (78.6%) had one or more congenitally missing teeth in the anterior region (cuspid to cuspid). Twenty-five out of 28 patients functioned well with their primary teeth and did not receive any prosthetic treatment yet. Three patients received prosthetic treatment before participating in the study (removable partial prostheses (*n* = 2); composite veneers in de lower front region (*n* = 1)). Twenty-three of the 29 asked controls agreed to participate. The characteristics of both groups are shown in Table 1. The prevalence of skeletal anomalies was comparable between the oligodontia (Angle class I: 35.7%, Angle class II: 64.3%, see Table 1) and control (Angle class I: 34.8%; Angle class II: 56.6%, see Table 1) group. With regard to the prevalence of skeletal malocclusion, again the prevalence of a deep bite was rather comparable between oligodontia patients (82.1%) and controls (69.6%), with the exception of microdontia which was only observed in oligodontia patients (57.1%).

Only the ‘graded response scale items applicable to everyone’ of the ChildQoLDAT were included in the OHrQoL analyses and therefore the following items were excluded: informative items (1a, 1b, 1d, 1e, 2d, 2e and 6a), Yes/No items (1c, 3b and 5j) and the ‘graded response scale items not applicable to everyone’ (2f and 5k) [12,13]. Two items were left unanswered by ≥15% of the oligodontia patients (2b and 3h) and these were excluded as well. The results of the included 26 items are shown in Table 2. Regarding ‘treatment’, the oligodontia patients felt that their treatment was more complicated than that of their friends (2a), hence this score was significantly lower than the controls. Also, dental appearance was scored worse by the oligodontia patients. Thus, oligodontia patients are more aware of and certainly worry about their appearance; for example, the scores of item 4a (“I feel embarrassed about the way my teeth look”) and 4f (“Most of my friends have teeth that look better than mine”) differed significantly than those of the controls. In contrast, eating, speaking, performing oral self-care and taking part in contact sports were not negatively influenced by having oligodontia (3a, 3c, 3d, 3e, 3f, 3g). Additionally, the oligodontia patients were not restricted in their social contact and/or by the reaction of other people (5a, 5b, 5c, 5d, 5e, 5f, 5g) and their treatment was supported by their family (5h).

Subgroup analyses revealed that older children with oligodontia (13–17 years) scored significantly lower than younger ones (11–12 years old), meaning that the oral health-related quality of life of the younger ones was less negatively influenced (*t*_26_ = 2.072, *p* = 0.048; Table 3). The total item score of the oligodontia group was not influenced by gender, number of congenitally missing teeth, front agenesis, orthodontic classification for occlusion, microdontia. Subgroup analyses were performed on the control group in terms of ‘age’ and ‘gender’ with an independent *t*-test and ‘orthodontic classification for occlusion’ with a one-way ANOVA. The control group analyses did not demonstrate any differences.

Patients could give a score of between 1 and 5 per item. The higher the score, the less negative the patient was about that item. Not all items were applicable (n.a.) to the controls. A Fisher’s Exact Test (95% CI) was performed to compare the results of the items that were completed by both the oligodontia patients and controls.

## 4. Discussion

The results of the condition-specific ChildQoLDAT show that the impact of oligodontia on the Dutch youngsters’ OHrQoL is limited and mainly concerns dental appearance and the complexity of the treatment. Remarkably, OHrQoL was significantly worse in patients above the age of 12 compared to younger patients. Compared to other chronically disorders in young patients with a similar age range, for instance temporomandibular joint arthritis affected by juvenile idiopathic arthritis, oligodontia seems to have less impact on OHrQoL [18].

According to the literature, it is presumed that the absence of several permanent teeth has a negative impact on the OHrQoL of those aged between 11–15 years [3,4]. However, the questionnaires used in previous OHrQoL studies do not give information about the exact problems experienced by children with oligodontia and about the impact of these problems. Considering the various items of this condition specific questionnaire, more insights have come to light about the condition specific problems in oligodontia. First, eating and speaking (in public) is not influenced by having oligodontia. This can be explained by the fact that most of these children have not lost their deciduous teeth yet. This is in agreement with the results of Laing et al., where only chewing difficulties were seen in patients who had lost their deciduous teeth without successors [5]. To investigate the impact of losing deciduous teeth (without successors) on the OHrQoL, only patients who lost their deciduous teeth should be included. However, the deciduous teeth are often maintained for many years (as there are no successors), and as a consequence the age range of the subjects would no longer fit with the age-criteria of the ChildQoLDAT (11–18 years). Therefore, investigating this factor was not considered feasible for this study. Secondly, it also seems that the patients are not too concerned about the reaction of other people, despite their consciousness of having oligodontia. Thus, the impact of the aforementioned items is limited, and this can be explained by the fact that most of these patients visit the centers for special dental care at an early age and they know that they will receive dental help for their missing permanent teeth. Conversely, oligodontia patients are concerned about their dental appearance and treatment complexity in comparison to their peers. This could be a consequence of becoming more aware about appearance during puberty at secondary school as older (≥13 years of age) oligodontia children had significantly lower (more negative) sores in comparison to the younger ones (11–12 years of age).

It would be interesting to compare the oligodontia group with a control group comprised of subjects not requiring orthodontic treatment. However, in the Netherlands, the majority of the children, non-oligodontia patients as well, receive orthodontic treatment. Hence, such a control group would be quite difficult to form. It may also introduce confounding factors such as dental anxiety, poor life style and financial limitations. As a compromise, a control group of children requiring orthodontic treatment (without congenitally absent teeth) was used.

Our results are not in line with Locker et al. and Wong et al., whereby both mentioned that most patients with congenitally missing teeth reported functional and psychosocial problems [3,4]. However, neither of the studies used a control group and this might have led to different conclusions [5].

In the Netherlands, children with oligodontia generally visit centers for special dental care at an early age, thus the potential negative effect on OHrQoL is probably limited. Aesthetic and functional concerns in children are tackled as much as possible and as soon as possible by (temporary) prosthodontic treatments, e.g., by restoring deciduous teeth and/or microdontic permanent teeth. Moreover, extra attention is given during the visits to oral self-care to preserve the deciduous teeth and thus prevent the presence of diastemas. The Dutch national health insurance scheme pays for the dental treatment of oligodontia patients which makes dental treatment of oligodontia available to all inhabitants. The impact of oligodontia on OHrQoL is probably greater in countries were dental treatment of oligodontia is not reimbursed.

The translated ChildQoLDAT was used for this study [12,13]. A disadvantage of this questionnaire is the negative formulation of almost all the items. For example, “I do not smile for photographs because of the way my teeth are”. This could make children more aware of problems which they had not been aware of before. The questionnaire was developed for children between the ages of 11–18 years and this restricts its use in long-term prospective studies. Besides, this makes the inclusion of more patients, also because of the low prevalence of oligodontia, difficult as was shown in the results of this study. Results from a recent study demonstrate that treatment of adolescent subjects affected by caries (restorations, endodontic treatment and extractions) improved OHrQoL [19]. For the research group of our study it is also interesting to investigate the impact of the treatment on the OHrQoL. Most items of the ChildQoLDAT are suitable for the comparison of scores before and after the orthodontic treatment, to study whether or not this treatment has improved the oral health-related quality of life. However, it is more interesting to measure the oral health-related quality of life after the whole long-term treatment, including definitive implant treatment, has finished. As most Dutch oligodontia patients receive dental implants after the age of 18, this particular questionnaire is not suitable for that propose. Additionally, the authors tried to create an unadulterated research group as much as possibly to limit the chance for selection bias. However, there is still a chance for selection bias as the selected patients came from different hospitals. For the present study, matching based on the severity of malocclusion was not considered feasible, as oligodontia patients are very heterogeneous from a dental and skeletal perspective [20]. As shown in the results three patients received prosthetic treatment. As these prosthodontic treatments were not permanent dental solutions, it was assumed that the impact of these treatments on OHrQoL would be limited.

Despite the small sample size and limitations of the ChildQoLDAT measure and the study, the results of this study are relevant as the condition specific items give more insight into the OHrQoL of patients with oligodontia prior to their orthodontic treatment.

## 5. Conclusions

The results of this study give more insight in the understanding of oligodontia on the OHrQoL in children. The impact of oligodontia on OHrQoL in youngsters is limited and mainly concerns dental appearance and the complexity of the treatment. Dentists should take the dental appearance in children diagnosed with oligodontia into consideration already at an early age (11 years).

## Figures and Tables

**Table 1:** Oligodontia and control group characteristics.

	Oligodontia Group (≥6 Congenitally Missing Teeth)	Control Group (No Congenitally Missing Teeth)
Number of patients	28	23
Gender (male/female)	14/14	15/8
Median age at questionnaire completion [IQR]	12 [11;13]	12 [11;13]
Orthodontic classification for occlusion (%)		
I	10 (35.7%)	8 (34.8%)
II	18 (64.3%)	13 (56.6%)
III	0 (0%)	2 (8.7%)

**Table 2:** Included item-scores (median, IQR) of the oligodontia and control patients. All questionnaires were completed prior to the onset of orthodontic treatment. CI = confidence interval; n.a. = not applicable.

Included Items		Oligodontia	Control	*p*-Value (95% CI)
	**Treatment**			
2a	I feel that my treatment is more complicated than the treatment my friends are having.	2 [1;3]	3 [3;4]	<0.000
2c	I’m worried about how my teeth will look at the end of my treatment.	4 [3;4]	4 [3;4]	0.815
	**Activities**			
3a	It takes me a lot longer to brush my teeth because of the gaps.	4 [4;4]	n.a.	
3c	Food gets stuck in the gaps between my teeth.	4 [2;4]	n.a.	
3d	Having missing teeth affects my speech, for example, I have a lisp or find it difficult to pronounce certain words.	4 [3;5]	n.a.	
3e	I do not eat in public places because of the way my teeth are.	5 [4;5]	5 [4;5]	0.777
3f	I am nervous about speaking aloud in public.	5 [4;5]	5 [4;5]	0.408
3g	I do not take part in contact sports because I worry about hurting my teeth.	5 [4;5]	5 [4;5]	0.769
	**Appearance**			
4a	I feel embarrassed about the way my teeth look.	3 [2;4]	4 [3;5]	0.023
4b	I do not smile for photographs because of the way my teeth are.	3 [2;5]	5 [4;5]	0.154
4c	I think my teeth look out of proportion, for example, they look too big or too small.	3 [2;4]	4 [3;5]	0.065
4d	The gaps in my teeth bother me.	3 [2;4]	n.a.	
4e	I worry about being left with a gap when my baby teeth fall out.	2 [2;3]	n.a.	
4f	Most of my friends have teeth that look better than mine.	2 [1;3]	3 [2;4]	0.010
4g	I do not laugh out loud with friends because of the way my teeth look.	4 [4;5]	5 [4;5]	0.242
4h	I worry about the size of the false teeth I will have fitted.	4 [4;5]	n.a.	
4i	I worry about the colour of the false teeth I will have fitted.	4 [4;5]	n.a.	
	**The Reaction of Other People**			
5a	I worry about how people will react to my missing teeth.	4 [3;4]	n.a.	
5b	I feel embarrassed about meeting people for the first time because of the way my teeth are.	4 [4;5]	5 [4;5]	0.053
5c	I wouldn’t want my friends to know I have missing teeth.	4 [4;5]	n.a.	
5d	People have commented on me having baby teeth.	4 [3;5]	n.a.	
5e	I worry that people might think it is weird if I have a false tooth.	4 [4;4]	n.a.	
5f	People laugh at me because of the way my teeth look.	4 [4;5]	5 [4;5]	0.103
5g	People have made me feel uncomfortable about the size of my teeth.	4 [4;5]	5 [4;5]	0.225
5h	My family supports the treatment I am having for my missing teeth.	5 [4;5]	n.a.	
5i	My family treat me differently because I have missing teeth, for example, they worry or are protective.	4 [4;5]	n.a.	

**Table 3:** Subgroup analyses of the total scores of the oligodontia patients’ included items (range 26–130).

	Subgroups	Total Score (Mean (SD))	*p*-Value (95% CI)
Gender	Male (*n* = 14)	93.2 (11.6)	0.282
	Female (*n* = 14)	98.1 (11.8)	
Age	11–12 years old (*n* = 19)	98.6 (11.4)	0.048
	13–17 years old (*n* = 9)	89.3 (10.3)	
Number of congenitally missing teeth	6–9 congenitally missing teeth (*n* = 13)	91.2 (9.0)	0.058
	≥10 congenitally missing teeth (*n* = 15)	99.5 (12.7)	
Tooth agenesis in front region	No missing anterior teeth (*n* = 6)	98.5 (6.7)	0.512
	Missing anterior teeth (*n* = 22)	94.9 (12.8)	
Orthodontic classification for occlusion	Class I (*n* = 10)	94.2 (12.3)	0.637
	Class II (*n* = 18)	96.4 (11.7)	
	Class III (*n* = 0)	n.a.	
Microdontia	No microdontia (*n* = 12)	97.8 (11.2)	0.421
	Microdontia (*n* = 16)	94.1 (12.3)	

The higher the score, the less negative the patient is about the OHrQoL. Independent *t*-tests (95% CI) were performed for all subgroup comparisons.
